# Effect of Physical Training on Exercise-Induced Inflammation and Performance in Mice

**DOI:** 10.3389/fcell.2021.625680

**Published:** 2021-02-04

**Authors:** Luiz Alexandre Medrado de Barcellos, William Antonio Gonçalves, Marcos Paulo Esteves de Oliveira, Juliana Bohnen Guimarães, Celso Martins Queiroz-Junior, Carolina Braga de Resende, Remo Castro Russo, Cândido Celso Coimbra, Albená Nunes Silva, Mauro Martins Teixeira, Barbara Maximino Rezende, Vanessa Pinho

**Affiliations:** ^1^Departamento de Ciências do Movimento Humano, Universidade do Estado de Minas Gerais (UEMG) – Unidade Ibirité, Ibirité, Brazil; ^2^Departamento de Morfologia, Instituto de Ciências Biológicas (ICB), Universidade Federal de Minas Gerais (UFMG), Belo Horizonte, Brazil; ^3^Hospital das Clínicas, Universidade Federal de Minas Gerais (UFMG), Belo Horizonte, Brazil; ^4^Departamento de Fisiologia e Biofísica, Instituto de Ciências Biológicas (ICB), Universidade Federal de Minas Gerais (UFMG), Belo Horizonte, Brazil; ^5^Laboratório de Inflamação e Imunologia do Exercício, Departamento de Educação Física, Escola de Educação Física da Universidade Federal de Ouro Preto, Ouro Preto, Brazil; ^6^Departamento de Bioquímica e Imunologia, Instituto de Ciências Biológicas (ICB), Universidade Federal de Minas Gerais (UFMG), Belo Horizonte, Brazil; ^7^Departamento de Enfermagem Básica, Escola de Enfermagem, Universidade Federal de Minas Gerais (UFMG), Belo Horizonte, Brazil

**Keywords:** physical training, muscular inflammation, neutrophil, exercise, oxidative stress

## Abstract

Acute exercise increases the amount of circulating inflammatory cells and cytokines to maintain physiological homeostasis. However, it remains unclear how physical training regulates exercise-induced inflammation and performance. Here, we demonstrate that acute high intensity exercise promotes an inflammatory profile characterized by increased blood IL-6 levels, neutrophil migratory capacity, and leukocyte recruitment to skeletal muscle vessels. Moreover, we found that physical training amplified leukocyte–endothelial cell interaction induced by acute exercise in skeletal muscle vessels and diminished exercise-induced inflammation in skeletal muscle tissue. Furthermore, we verified that disruption of the gp-91 subunit of NADPH-oxidase inhibited exercise-induced leukocyte recruitment on skeletal muscle after training with enhanced exercise time until fatigue. In conclusion, the training was related to physical improvement and immune adaptations. Moreover, reactive oxygen species (ROS) could be related to mechanisms to limit aerobic performance and its absence decreases the inflammatory response elicited by exercise after training.

## Introduction

Inflammation is a natural process of mammalian immune responses to tissue damage, triggered by invading pathogens or sterile tissue injury. This response comprises temporally and spatially orchestrated events in which inflammatory cells and mediators act to interrupt harmful stimuli and induce tissue repair, promoting a return to homeostasis (Medzhitov, 2010; Alessandri et al., 2013). Exercise-induced inflammation has been shown to be an essential for restoring homeostasis and improving physical capacity (Deyhle et al., 2015). During exercise, muscle resident cells and vascular endothelium release inflammatory mediators that generate chemotactic signals, which drive leukocyte trafficking to muscular tissue (Kolaczkowska and Kubes, 2013). The presence of these cells is associates with loss of muscular architecture after exercise (Ogilvie et al., 1988; Hortobagyi et al., 1998). This effect could be induced by metabolic processes characterized by events involving oxidative stress (Barbieri and Sestili, 2012).

Oxidative stress induced by reactive oxygen species (ROS) is increased by muscular contraction (Jackson et al., 2007) and is able to prolong muscular inflammation induced by acute exercise. Several studies have indicated that ROS produced during the exercise are required to activate signal transduction pathways in inflammation mechanisms and have an essential role in the physiological adaptive process in muscle cells (Barbieri and Sestili, 2012). An increase in antioxidant capacity through the intake of nutritional supplements appears to reduce exercise-induced muscle damage and may improve performance and recovery (Child et al., 1999; Taherkhani et al., 2020). However, whether training is able to provoke an adaptation in the inflammatory response induced by exercise and whether ROS are important to this process remain unclear. Thus, the present study aimed to assess the influence of physical training on inflammation and physical performance and the role of ROS in this process.

## Materials and Methods

### Ethics Statement

The animal care and handling procedures were in accordance with the guidelines of the Institutional Animal Care and Use Committee, and the study received prior approval from Animal Ethics Committee of Universidade Federal de Minas Gerais (UFMG; protocol 7412/2012).

### Mice

Eight to twelve weeks-old wild-type C57BL/6 male mice were obtained from the Centro de Bioterismo (UFMG) and gp91^phox^ knockout C57BL/6 male mice were provided from Jackson Farms (Glensville, NJ, United States) and maintained at our laboratory. All mice were housed under standard conditions in a temperature-controlled room (23 ± 1°C) on an automatic 12 h/12 h light/dark cycle and had free access to commercial rodent food and water.

### Running Treadmill Familiarization

Running treadmill familiarization was performed on different treadmills (LE400, Panlab, Harvard Apparatus, Cornella, Spain or Gaustec Magnetismo, Nova Lima, MG, Brazil) according to each design as previous protocols (Lacerda et al., 2005; Primola-Gomes et al., 2007).

### Incremental-Speed Running Test Until Fatigue

An incremental-speed running test until fatigue on a treadmill (LE400, Panlab, Harvard Apparatus, Cornella, Spain) was performed to assess the peak speed (S_peak_). All these incremental running tests were performed at least 24 h after the last familiarization session. During this test, the mice began running at 6 m min^–1^ and the speed was increased by 3 m min^–1^ every 3 min at 5° slope until fatigue (Ferreira et al., 2007). Fatigue was defined as the moment at which the animals were unable to maintain their pace with the treadmill speed for at least 10 s, even when exposed to slight electrical stimulation.

### Rest and Low or High Intensity Exercise

Wild-type C57BL/6 male mice were allocated into three groups, rest, low intensity (40% of S_peak_), or high intensity (80% of S_peak_) of exercise on the treadmill to analyze oxygen consumption (VO_2_) (LE400, Panlab, Harvard Apparatus, Cornella, Spain). The exercise intensity was calculated from S_peak_ achieved in the incremental speed-running test until fatigue. Each exercise or rest lasted 30 min. The slope of the treadmill was maintained at 5°. All these procedures were performed at least 48 h after the incremental-speed running test until fatigue.

### Fixed-Speed Running Test Until Fatigue

The workload and the VO_2_ of the mice at fixed-speed running test until fatigue on treadmill was evaluated (LE400, Panlab, Harvard Apparatus, Cornella, Spain) pre (at least 48 h after incremental-speed running test until fatigue) and post 4 weeks aerobic training (at least 48 h after the last training session). The intensity of this test corresponded to 80% of the S_peak_ achieved at incremental-speed running test until fatigue and the treadmill slope adopted was also 5°. The workload attained in the fixed-speed running test was used as a reference for the load prescription in the exercise sessions throughout the aerobic training.

### Physical Training Protocol

In all procedures involving training the mice were randomized allocated into sedentary or trained experimental groups. The trained group was submitted to exercise on a treadmill (Gaustec Magnetismo, Nova Lima, MG, Brazil) across 4 weeks, with 5 weekly sessions always performed at the same time of the day (8:00 a.m. to 10:00 a.m.). The slope of the treadmill was maintained at 5° throughout the training protocol. To ensure similar handling and exposure for each treadmill setup, the sedentary group mice performed an exercise at a speed of 6 m min^–1^ with maximal duration of 5 min, which was adjusted daily according to the body mass of the mice. The aerobic training load from the first to the last week was equivalent to 60, 70, 80, and 90% of the workload (%W) performed in the fixed-speed running test until fatigue prior to the 4 weeks aerobic training.

### VO_2_ Measurement

VO_2_ was measured via open-flow indirect calorimeter (LE400, Panlab, Harvard Apparatus, Cornella, Spain) calibrated with a certified gas mixture (high O_2_ = 50.05%, high CO_2_ = 1.51%, low O_2_ = 20.02% and low CO_2_ = 0.00%). The air flow rate established was equivalent to 0.6 L min^–1^ throughout the procedures. VO_2_ data were analyzed using a computerized system (Metabolism software version 2.2.01 Panlab, Harvard Apparatus), transformed to milliliters per minute and relativized by the mice body mass (mLO_2_ kg^–0^.^75^ min^–1^).

### Body Mass

The percentage of the body mass variation was calculated by the difference between the weight of the mouse before the second test (post training) and the weight before the first test (pre training) divided by the pre training weight, multiplied by 100.

### Assessment of Pulmonary Mechanics

Pulmonary dysfunction was measured as we previously described (Campa et al., 2018; Russo et al., 2018). For invasive *in vivo* assessment, mice were anesthetized and tracheostomized, then were placed in a whole-body plethysmograph to maintain spontaneous breathing connected to a computer-controlled ventilator (Forced Pulmonary Maneuver System^®^, Buxco Research Systems©, Wilmington, North Carolina, United States). Under mechanical respiration the Dynamic Compliance (Cdyn) and Lung Resistance (Rl) were determined by Resistance and Chord Compliance RC test. To measure the Inspiratory Capacity (IC) a Pressure-Volume maneuver was performed, which inflates the lungs to a standard pressure of +30 cm H_2_O and then slowly exhales until a negative pressure of −30 cm H_2_O is reached. To evaluate airway hyperresponsiveness (AHR), the same mice used in previous maneuvers (basal condition) received Methacholine, 1 mg Kg^–1^ (Acetyl-β-methylcholine chloride, A-2251, Sigma-Aldrich St. Louis, MO, United States) i.v. and after 10 s, a new set of maneuvers were conducted to assess Rl changes. Suboptimal maneuvers were rejected and for each test at least three acceptable maneuvers were conducted in every single mouse to obtain a reliable mean for all numeric parameters.

### Intravital Microscopy

The mice were anesthetized, and the femoral straight muscle venules were exposed by a resection in the anterior part of the thigh for exhibition of the femoral straight muscle. The animals received an i.v. injection from Rodamin 6G (0.3 mg kg^–1^, Sigma-Aldrich, Germany) to fluorescent labeling of leukocytes. An intravital microscope (ECLIPSE 50i; Nikon) with a 20 objective lens was used to examine the muscle microcirculation. A digital camera (DS-Qi1MC; Nikon) was used to acquire the images that were recorded for playback analysis with Nikon imaging software. The counting of rolling and adherent leukocytes was realized according to our previously published method (Rezende et al., 2017). Rolling leukocytes was defined as those cells moving thought the observed field at a velocity less than that of erythrocytes within a given vessel during 1 min. Leukocyte was considered to be adherent if it remained stationary for at least 30 s, and total leukocyte adhesion is quantified as the number of adherent cells within a 100 μm length of venule in 1 min.

### Quantification of Cytokines

Hundred milligrams of the quadriceps muscle (wet) were separated and homogenized with PBS containing antiproteases (0.1 mM PMSF, 0.1 nM hydrochloric benzethonium, 10 mM EDTA and 20 Ki aprotinin A) and 0.05% Tween 20. The samples were centrifuged for 10 min, at 10,000 rpm and at 4°C. The supernatant was used for the ELISA assay with a 1: 4 dilution. The ELISA assay was performed according to the manufacturer’s instructions (R&D System) and quantified from the 492 nm wavelength acquired in a plate reader (Spectramax plus 384, Molecular Devices, United States).

### Histology

Left femoral quadriceps was sectioned transversely in half, with the proximal half placed in O.C.T (Tissue-Tek^®^; Sakura, Netherlands) at −25°C. The tissues were sliced at 10 μm thickness using a cryostat (Leica CM1850, Leica Biosystems, Germany), immediately placed on silanized slide and fixed for 1 h in acetone at −80°C. The tissue sections were stained with HE according to standard histological technique and evaluated histologically in a blinded manner. The following parameters were evaluated and classified as absent, mild, moderate, or intense: fiber atrophy (morphological alteration), muscle necrosis/degeneration, inflammatory infiltrate in the endomysium/perimysium, endomysium/perimysium distension. These criteria were first described by Rizo-Roca et al. (2015).

### Neutrophil Chemotaxis Assays

Neutrophil chemotaxis assay was performed using a modified Boyden chamber (Neuroprobe, Pleasanton, United States) and polycarbonate filters (4 μm; Neuroprobe, Pleasanton, United States) as previously described by Tavares-Murta et al. (2002). Bone marrow neutrophils were isolated and submitted to chemotactic stimulus with 28 μL of N-formyl-methionyl-leucyl-phenylalanine (fMLP). On one side of the membrane, the fMLP was placed, and on the other, a suspension of 1.0 × 10^6^ neutrophils/mL. After 60 min of incubation at 37°C, the filter was removed, washed and fixed in methanol. Then, the membranes were stained in panotic for microscopic counting. Five random fields per sample were selected on the membrane to count cell migration.

### Statistical Analysis

Data are expressed as mean ± SEM. The normal distribution was verified by the Shapiro-Wilk test. Comparisons among the groups were performed by unpaired Student’s *t*-test, one-way ANOVA or two-way ANOVA followed by the Tukey or Sidak *post hoc* analysis, whenever applicable. Statistical significance was set as *p* < 0.05. The statistical package used was GraphPad Software’s Prism 6^®^.

## Results

**FIGURE 1:**
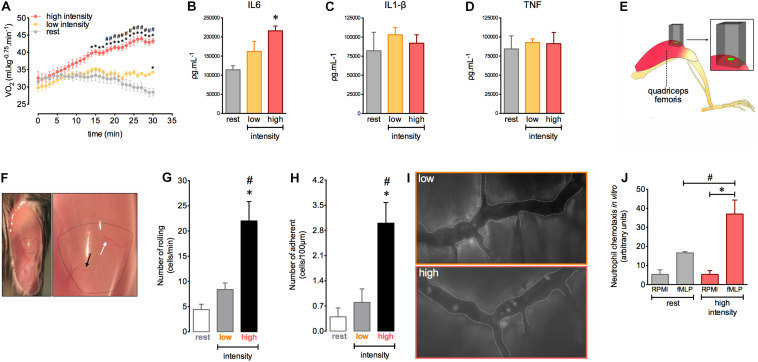
Homeostasis disruption by exercise induces leukocyte recruitment and late alteration of IL-6 balance in quadriceps muscles. VO_2_ immediately before and during 30 min at rest (*n* = 5) or at low (*n* = 5) and at high (*n* = 6) intensity exercises **(A)**. IL-6 **(B)**, IL-1β **(C)**, and TNF **(D)** levels in the quadriceps femoris 12 h after rest (*n* = 4) or low (*n* = 4) and high intensity (*n* = 4) exercises. Neutrophil chemotaxis by Boyden chamber assay. Bone marrow isolated-neutrophils (1 × 10^6^ cells/ml) from rest (*n* = 3) or high intensity (*n* = 3) groups stimulated by fMLP (28 μl) **(E)**. The light beam from intravital microscopy targeting the distal region of the quadriceps femoris for intravital analysis **(F)**. Exposed quadriceps for intravital experiment **(G)**. The most (black arrows) and least (white arrows) frequently accessed vessels in the delimited region of the quadriceps. Leukocyte rolling **(H)** and adhesion **(I)** on microvasculature 12 h after rest (*n* = 5), low (*n* = 5) or high intensity (*n* = 6). Representative figure showing the vessel (dotted white line) and leukocytes (luminous bodies) quantified in **(H–J)**. Data are expressed as mean ± SEM; ^∗^*p* < 0.05 compared to: rest **(A,B,H,I)** and RPMI high intensity group **(E)** and ^#^*p* < 0.05 compared to: low intensity group **(A,H,I)** and fMLP rest group **(E)** using two-way ANOVA **(A)** or one-way ANOVA **(B–E,H,I)** followed by Tukey *post hoc* analysis.

The magnitude and amount of stress induced by movement are important factors for morphological and functional changes in the body. We first determined the exercise intensity required to induce an efficient acute response during a single exercise session consisting of running on a treadmill. We observed that high intensity exercise (80% of the S_peak_) triggered an aerobic demand that increased in a time dependent manner. This was observed as a gradual increase in oxygen consumption (VO_2_) during the course of exercise (Figure 1A). Low exercise intensity (40% of the S_peak_) was unable to induce significant changes in VO_2_ (Figure 1A). Moreover, we found increased muscular levels of IL-6 after 12 h in mice subjected to high intensity exercise (Figure 1B). The levels of IL1-β and TNF-α were similar in all groups (Figures 1C,D).

Given the importance of the immune response during physical training, we next evaluated the interactions between leukocytes and muscle microvasculature after a single exercise session at different intensities. We assessed a specific distal region of the quadriceps femoris, localized near the rectus femoris tendon (Figure 1E). To ensure reproducibility and to reduce variability during measurements, we chose two vessels in this muscle region to better track the vessels during intravital microscopy analysis (Figure 1F). We observed an increase in leukocyte rolling and adhesion in the muscular vessels after 12 h in the group subjected to high intensity exercise (Figures 1G–I). We also found increased neutrophil chemotaxis toward chemotactic factor fMLP *ex vivo* using cells purified from the bone marrow of exercised mice (Figure 1J). Together, these results indicate that high intensity exercise applied by running on a treadmill offers the optimal conditions to provoke an important inflammatory response in muscular tissue.

**FIGURE 2:**
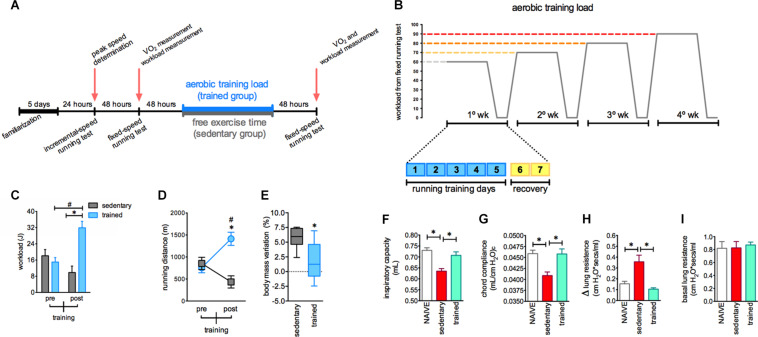
The aerobic training changes body mass gain dynamics, aerobic performance and respiratory mechanics in mice. Schematic diagram for experimental approach visualization **(A)**. Aerobic training load characteristics **(B)**. Workload **(C)** and running distance **(D)** evaluated pre and post aerobic training in both sedentary (*n* = 7) and trained (*n* = 8) groups. Body mass variation in sedentary and trained groups **(C)**. Inspiratory capacity **(F)**, chord compliance **(G)**, lung resistance **(H)**, and basal lung resistance **(I)** in the naive (*n* = 8), sedentary (*n* = 6) and trained groups (*n* = 8) evaluated 72 h after the fixed-speed running test until fatigue post training. Data are expressed as mean **(C,D,F–I)** or min to max in the box plot graph **(E)** ± SEM. ^∗^*p* < 0.05 compared to: sedentary post **(C)**; trained pre **(D)**; sedentary **(E–I)** and ^#^*p* < 0.05 compared to: trained pre **(C)**; sedentary post **(D)** using two-way ANOVA following by Sidak *post hoc* analysis **(C,D)**, two-tailed *t*-test **(E)** or one-way ANOVA followed by Tukey’s *post hoc* analysis **(F–I)**.

Our experimental design (Figure 2A) was able to induce several adaptations in mice. Of note, the proposed aerobic training protocol offered a gradual increase in the load based on a targeted workload for each training session (Figure 2B, for more details see section “Materials and Methods”). Here, this experimental approach was efficient to elicit consistent change in mice aerobic status. We observed that mice increased the workload and total running distances in aerobic tests after the training period (Figures 2C,D, respectively). Moreover, the body mass variation in trained mice was less pronounced compared to the sedentary group (Figure 2E). These consistent alterations in aerobic status and control of the increase in body mass were accompanied by enhanced lung function in response to stressful exercise. We observed that 72 h after a single exercise session under fatigue conditions, the sedentary mice group displayed reduced inspiratory capacity and compliance (Cchord) in response to stressful exercise and an increased sensitivity to bronchoconstriction evoked by methacholine injection. However, the trained mice did not present this impact of stressful exercise, showing preserved inspiratory capacity and compliance accompanied by reduced airway hyper reactivity induced by methacholine (Figures 2F–H, respectively). No changes in the basal resistance were observed (Figure 2I).

**FIGURE 3:**
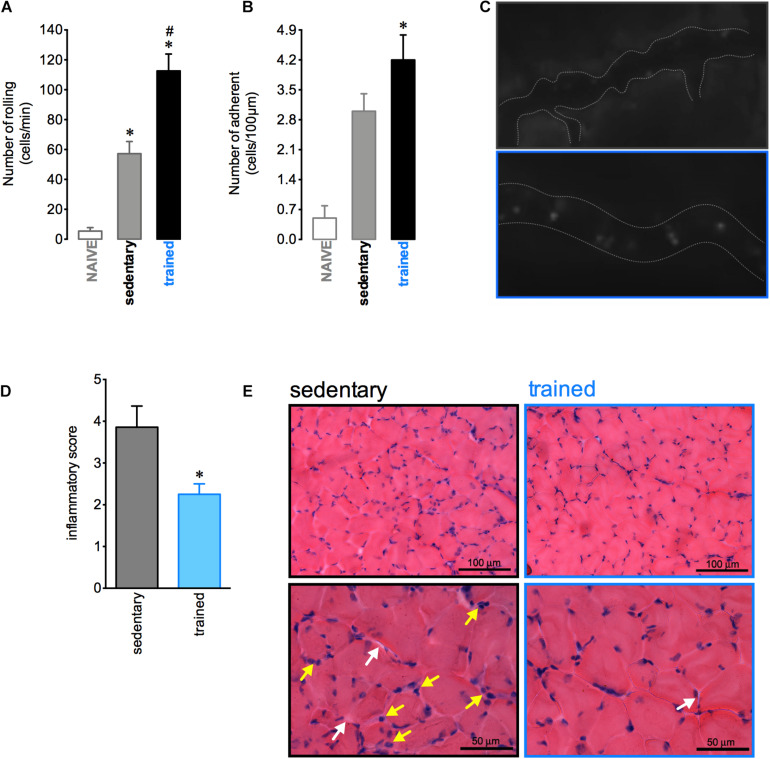
Aerobic training changes the inflammatory response in the muscle. Quantification of leukocyte rolling **(A)** and adhesion **(B)** on microvasculature of the naive (*n* = 6), sedentary (*n* = 5), and trained (*n* = 6) mice 12 h after the fixed-speed running test until fatigue post training. Representative figure showing the vessel (dotted white line) and leukocytes (luminous bodies) quantified in (**A–C)**. Histopathological quantification **(D)** and representative histological slide from H&E staining **(E)** of muscular tissue samples from sedentary (*n* = 7) or trained (*n* = 5) mice 72 h after the fixed-speed running test until fatigue post training. The yellow and white rows represent the inflammatory cells and endomysium distension, respectively. Data are expressed as mean ± SEM; ^∗^*p* < 0.05 compared to: naive **(A,B)** or sedentary group **(D)** and ^#^*p* < 0.05 compared to: sedentary group **(A)** using one-way ANOVA followed by Tukey’s *post hoc* analysis **(A,B)** or two-tailed *t*-test **(C,D)**.

The aerobic training protocol was able to induce inflammatory response adaptations in muscular tissue. After 12 h of the fixed-speed running test there was an increase in both rolling and adhesion of leukocytes in muscle vessels of trained mice (Figures 3A–C). Nevertheless, 72 h after this test the loss in muscular architecture and the increase of inflammatory cells were more pronounced in sedentary than trained mice (Figures 3D,E). These results indicated that the profile of inflammatory response induced by exercise exhibited by trained mice could be important to the subsequent recovery of muscular tissue. Moreover, we demonstrated that trained mice presented a slight alteration in cellular profile with an increase in the blood CD8 + lymphocytes and a decrease in the bone marrow macrophages F4/80 + (Supplementary Figures 1D,G, respectively). We not observed any alterations in other leukocytes recovered in either blood (Supplementary Figures 1A–C) or bone marrow (Supplementary Figure 1F,H,I).

**FIGURE 4:**
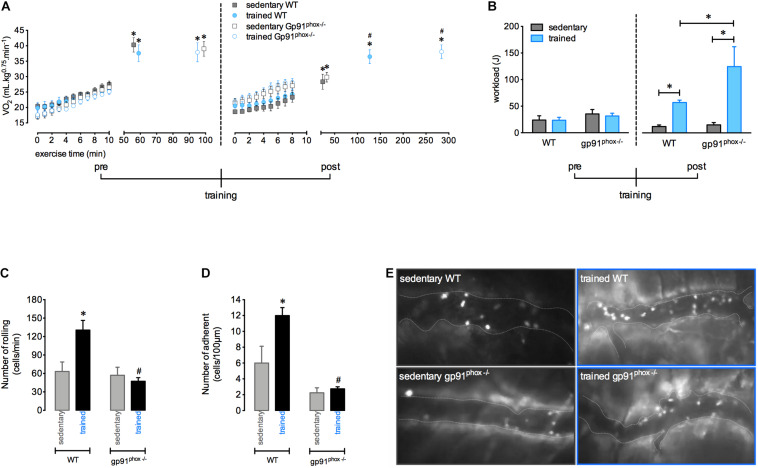
The absence of ROS from NADPH-oxidase increases running capacity and abrogates the greater exercise-induced leukocyte interaction on vasculature after aerobic training. VO_2_ immediately before and during the fixed-running test until fatigue in the wild-type (sedentary, *n* = 7 and trained, *n* = 6) and gp91^phox^^–/–^ (sedentary, *n* = 5 and trained, *n* = 4) groups, pre and post aerobic training **(A)**. Workload pre- and post-aerobic training in all groups **(B)**. Leukocytes rolling **(C)** and adhesion **(D)** on microvasculature 12 h after the fixed-speed running test until fatigue in all groups. Representative figure showing the vessel (dotted white line) and leukocytes (luminous bodies) quantified in (**C–E)**. Data are expressed as mean ± SEM; ^∗^*p* < 0.05 compared to: 0 min **(A)** and respective sedentary groups **(B–D)** and ^#^*p* < 0.05 compared to: respective sedentary **(A)** and respective trained groups **(C,D)** using two-way ANOVA followed by Tukey’s *post hoc* analysis.

To verify the influence of ROS production on aerobic status after training and in leucocyte interactions with the muscular vasculature after exercise, we used gp91^phox–/–^ mice, which are animals with non-functional NADPH-oxidase. Trained mice showed a higher VO_2_ at the moment of fatigue than sedentary mice. Furthermore, it was observed that physical training improved the time to fatigue in both WT and gp91^phox–/–^ as demonstrated by the higher workload achieved in the fixed-running test compared to the respective sedentary mice. However, exercise interruption post training was delayed for gp91^phox–/–^ trained mice compared to WT trained mice (Figure 4A). In addition, the lack in ROS production potentializes the improvement in the running performance induced by aerobic training, which could be evidenced by the superior increase of workload realized by gp91^phox–/–^ trained mice compared to workload of WT trained mice (Figure 4B). In contrast, the increase of rolling and adhesion expected 12 h after a single exercise session was inhibited in trained mice with a deficiency of ROS production (Figures 4C–E).

## Discussion

Although it has been established that a single session of intense exercise is able to induce a considerable local and systemic inflammatory response, which could be reduced by short period of exercise repetition (Suzuki et al., 1996, 1999), little is known about the chronic effects of the training process on exercise-induced inflammation. The results found in this study can be summarized by the following points: acute high intensity exercise induced an important inflammatory response in the muscular tissue as demonstrated by (1) increases in the level of IL-6 in muscle, the amplified number of rolling and adhesion cells in the quadriceps vessels, and the stimulation of neutrophil chemotactic activity isolated from bone marrow in exercised mice. Moreover, we developed an effective running training for mice that was able to induce several adaptations, including (2) elevated workload and running distances reached in aerobic tests after the training period accompanied by reduced gains in body mass; (3) enhanced lung function; (4) high number of rolling and adherent cells on muscle vessels, but preserved quadriceps muscular architecture with a predominance of mononuclear cells. Finally, the deficiency in ROS production by NADPH oxidase enzyme (5) potentialized the improvement in the running performance induced by physical training, and (6) diminished the rolling and adhesion cells related to exercise.

In this work, the intensity of exercise was decisive in stimulating the muscle IL-6 synthesis assessed 12 h post exercise. Muscle production of IL-6 is related to the exercise mode, intensity, and duration (Nieman et al., 1998; Ostrowski et al., 2001). Muscle-produced IL-6 may act on skeletal muscle, in a paracrine manner, by regulating myogenesis, glucose uptake and use of glycogen by the contracting muscle and also exert systemic effects such as increased hepatic glucose production and lipolysis, when released into the circulation (Fischer, 2006; Serrano et al., 2008). Circulating IL-6 normally increases immediately in response to contracting muscle and declines in the post-exercise period (Suzuki et al., 2002). Raised plasma IL-6 after exercise may a play role in neutrophil mobilization into the circulation (Yamada et al., 2002). In fact, the kinetics of IL-6 may be different depending on whether there was muscle damage induced by exercise (Febbraio and Pedersen, 2002; Petersen and Pedersen, 2006). Thus, it is suggested that the highest level of IL-6 in the muscle 12 h after the exercise event could originate from immune cells present in the damaged muscle during high-intensity exercise. We also observed that acute high intensity exercise induced elevation of the number of rolling and adherent cells in the muscle vasculature. It is well established that prolonged exercise is associated with high levels of oxidative stress that stimulate the inflammatory response. This inflammation may result from muscle damage triggered by mechanical and metabolic exercise loads (Barbieri and Sestili, 2012). Previous notable results from our group also showed the interaction of neutrophils with endothelial cells in quadriceps muscles after exercise until fatigue by using intravital microscopy, corroborating our findings. We found that high intensity exercise stimulated the *ex vivo* migratory capacity of neutrophils isolated from bone marrow 12 h post-exercise. The migration of neutrophils from bone marrow to the circulation is influenced by stress hormones, G-CSF and cytokines released during exercise (Suzuki et al., 2000; Yamada et al., 2002; Summers et al., 2010). Additionally, it has been showed that aerobic exercise mobilizes hematopoietic stem cells in an intensity-dependent manner (Baker et al., 2017). However, the precise mechanisms by which exercise inflammation leads to hematopoiesis and consequently neutrophilia still need to be better elucidated.

We demonstrated an effective protocol of running training for mice that was able to improvement of physical capacity and pulmonary function, including inspiratory capacity, chord compliance, and lung resistance. It has been previously shown that pulmonary capability is correlated with exercise tolerance (Kerti et al., 2018) and it may be associated with our results from the elevated workload and running distances reached in the aerobic tests after the training period.

Interestingly, although we observed a greater number of rolling and adherent cells in trained mice compared to sedentary mice, this result was accompanied by a lower inflammatory score in the quadriceps of the trained group, with a predominance of mononuclear cells. Usually, neutrophils infiltrate the extracellular space around the damage, peaking between 6 and 24 h after exercise. Subsequently, there is an increase of pro-inflammatory macrophages within 72 h followed by an influx of macrophages with anti-inflammatory and pro-myogenic phenotypes, which may remain for more than 6 days after damage (Saini et al., 2016). In the present study, reduced muscular inflammation infiltration and endomysium distension indicate less tissue damage, which may be related to mononuclear cells with anti-inflammatory properties, but the profile and role of these cells in muscle repair were not investigated here. Exercise is accepted as an anti-inflammatory therapy in inflammatory diseases such as cardiovascular disease, diabetes, and Alzheimer’s (Pedersen and Saltin, 2015). The mechanisms by which exercise training induces these anti-inflammatory effects remains unclear, but there are several intriguing possibilities, including release of endogenous products, such as heat shock proteins, selective reduction of visceral adipose tissue mass or reduction of infiltration of adipocytes by macrophages, shifts in immune cell phenotype, cross-tolerizing effects, or exercise-induced shifts in accessory proteins of toll-like receptor signaling (Petersen and Pedersen, 2006; Flynn et al., 2007). It is also important to assess whether the arrival dynamics of cell subtypes is modified by physical training, as monocytes, which could orchestrate inflammation resolution, repair, and increase muscle performance. This possibility deserves further investigation in the future.

In a previous study published by our group pharmacological blockade (apocynin) or genetic deletion of NADPH-oxidase (gp91^phox–/–^ mice) inhibited leukocytes recruitment after a single fatiguing exercise. Furthermore, the reduction of ROS production by apocycnin prevented the exercise-induced adhesion molecules (E-selectin, L-selectin and PECAM) expression, which may explain the increasing in the numbers of rolling, adherent, and transmigrating neutrophil, showing the role of ROS from NADPH-oxidase in the skeletal muscle inflammation process (Nunes-Silva et al., 2014). Thus, ROS may regulate cell adhesiveness to the vessel wall of the exercised muscle. ROS- mediated leukocytes recruitment after exercise may also contribute to the muscle remodeling signaling, including angiogenesis, hypertrophic response and mitochondrial biogenesis (Gomes et al., 2012). Similarly, using the genetically modified animals gp91^phox–/–^, we demonstrated that deficiency of ROS is related to a lower number of rolling and adherent leukocytes in the muscle vessels after training. Moreover, the gp91^*phox*–/–^ mice presented an exacerbation in the increase of the workload induced by training. This result suggests that the chronic absence of ROS could impair a probable role of exercise-induced inflammation in controlling fatigue mechanisms and perhaps expose the organism to risks related to excessive exercise. Thus, the importance of investigations on the use of antioxidant strategies for exercise performance is highlighted. In fact, several studies have explored the effectiveness of antioxidant supplementation to enhance performance and adaptation to training, but its benefits and risks remain unknown (Bjornsen et al., 2016; Rothschild and Bishop, 2020).

In conclusion, acute high intensity exercise induced an evident inflammatory response. Additionally, the training was related to physical improvement and immune adaptations. Finally, our data suggest that ROS could be related to maintaining signaling for the limits of aerobic physical performance adaptation to training through the increased inflammatory response elicited by exercise. Nevertheless, the role of oxidative balance on aerobic performance and exercise-induced inflammation after training still needs to be elucidated.

## Data Availability Statement

The original contributions presented in the study are included in the article/Supplementary Material, further inquiries can be directed to the corresponding author/s.

## Ethics Statement

The animal study was reviewed and approved by Animal Ethics Committee of Universidade Federal de Minas Gerais (UFMG; protocol 7412/2012).

## Author Contributions

LB: data collection, analysis of the results, and writing of the manuscript. WG: data collection, writing of the manuscript, and built the figures. ME and CR: data collection. JG: assisted during interpretation of the results and writing of the manuscript. CQ-J: microscopy analysis and interpretation of the results. RR: pulmonary data collection and interpretation of the results. CC: assisted during the exercise data collection and availability of the laboratory. AS: assisted during the project planning, writing of the manuscript, and the data collection. MT: assisted during the project planning and financial support. BR and VP: assisted during the project planning, data collection, and supervise this manuscript. All authors contributed to the article and approved the submitted version.

## Conflict of Interest

The authors declare that the research was conducted in the absence of any commercial or financial relationships that could be construed as a potential conflict of interest.

## Supplementary Material

Supplementary Figure 1 Effect of aerobic training on the profile of immune cells. The percentage of neutrophil Ly6G^+^, macrophage F480^+^, TCD4^+^ and TCD8^+^ cells of the sedentary (*n* = 7) and training (*n* = 8) mice were evaluated in blood (**A–D**, respectively) and bone marrow (**F–I**, respectively) by flow cytometry 72 h after fixed-speed running test until fatigue post training. Representative dot plots illustrating neutrophil Ly6G^+^, macrophage F480^+^, TCD4^+^, and TCD8^+^ cells of the sedentary (upper panel) and trained (below panel) mice in the blood **(E)** and bone marrow **(J)**. Data are expressed as mean ± SEM; ^∗^*p* < 0.05 compared to sedentary using two-tailed *t*-test. Cells from bone marrow and blood were plated, 1 × 10^6^ cells/well, in a 96-well plate and stained for extracellular molecular expression patterns using mAbs against mouse CD3 (Alexa-488 conjugated), CD4 (APC conjugated), CD8 (PE conjugated), CD11b (Alexa-488 conjugated), F4/80 (PE conjugated), and Ly6G (APC conjugated) (from BD Pharmingen, Le Pont de Claix, France). The cells were incubated with 20 μl/well of antibody solution 30’/4°C, followed by fixation in 4% of paraformaldehyde. Limits for the quadrant markers were always set based on negative populations and isotype control antibodies. The frequency of positive cells was analyzed by FlowJo X v10.2 software, using a gate that included lymphocytes, neutrophils and/or macrophages. The frequency (percentage) of the analyzed population in the total acquired events was used in the construction of the graphs.

## References

[B1] Alessandri A. L. Sousa L. P. Lucas C. D. Rossi A. G. Pinho V. Teixeira M. M. (2013). Resolution of inflammation: mechanisms and opportunity for drug development. *Pharmacol. Ther.* 139 189–212. 10.1016/j.pharmthera.2013.04.006 23583354

[B2] Baker J. M. Nederveen J. P. Parise G. (2017). Aerobic exercise in humans mobilizes HSCs in an intensity-dependent manner. *J. Appl. Physiol.* 122 182–190. 10.1152/japplphysiol.00696.2016 27881669 PMC5283849

[B3] Barbieri E. Sestili P. (2012). Reactive oxygen species in skeletal muscle signaling. *J. Signal. Transduct.* 2012:982794. 10.1155/2012/982794 PMC3235811 22175016

[B4] Bjornsen T. Salvesen S. Berntsen S. Hetlelid K. J. Stea T. H. Lohne-Seiler H. (2016). Vitamin C and E supplementation blunts increases in total lean body mass in elderly men after strength training. *Scand. J. Med. Sci. Sports* 26 755–763. 10.1111/sms.12506 26129928

[B5] Campa C. C. Silva R. L. Margaria J. P. Pirali T. Mattos M. S. Kraemer L. R. (2018). Inhalation of the prodrug PI3K inhibitor CL27c improves lung function in asthma and fibrosis. *Nat. Commun.* 9:5232. 10.1038/s41467-018-07698-6 PMC6290777 30542075

[B6] Child R. Brown S. Day S. Donnelly A. Roper H. Saxton J. (1999). Changes in indices of antioxidant status, lipid peroxidation and inflammation in human skeletal muscle after eccentric muscle actions. *Clin. Sci.* 96 105–115. 10.1042/cs0960105 9857113

[B7] Deyhle M. R. Gier A. M. Evans K. C. Eggett D. L. Nelson W. B. Parcell A. C. (2015). Skeletal muscle inflammation following repeated bouts of lengthening contractions in humans. *Front. Physiol.* 6:424. 10.3389/fphys.2015.00424 26793125 PMC4709832

[B8] Febbraio M. A. Pedersen B. K. (2002). Muscle-derived interleukin-6: mechanisms for activation and possible biological roles. *FASEB J.* 16 1335–1347. 10.1096/fj.01-0876rev 12205025

[B9] Ferreira J. C. Rolim N. P. Bartholomeu J. B. Gobatto C. A. Kokubun E. Brum P. C. (2007). Maximal lactate steady state in running mice: effect of exercise training. *Clin. Exper. Pharmacol. Physiol.* 34 760–765. 10.1111/j.1440-1681.2007.04635.x 17600553

[B10] Fischer C. P. (2006). Interleukin-6 in acute exercise and training: what is the biological relevance? *Exerc. Immunol. Rev.* 12 6–33. 17201070

[B11] Flynn M. G. Mcfarlin B. K. Markofski M. M. (2007). The anti-inflammatory actions of exercise training. *Am. J. Lifestyle Med.* 1 220–235. 25431545 10.1177/1559827607300283 PMC4243532

[B12] Gomes E. C. Silva A. N. De Oliveira M. R. (2012). Oxidants, antioxidants, and the beneficial roles of exercise-induced production of reactive species. *Oxid. Med. Cell Longev.* 2012:756132. 10.1155/2012/756132 PMC3372226 22701757

[B13] Hortobagyi T. Houmard J. Fraser D. Dudek R. Lambert J. Tracy J. (1998). Normal forces and myofibrillar disruption after repeated eccentric exercise. *J. Appl. Physiol.* 84 492–498. 10.1152/jappl.1998.84.2.492 9475858

[B14] Jackson M. J. Pye D. Palomero J. (2007). The production of reactive oxygen and nitrogen species by skeletal muscle. *J. Appl. Physiol.* 102 1664–1670. 10.1152/japplphysiol.01102.2006 17082364

[B15] Kerti M. Balogh Z. Kelemen K. Varga J. T. (2018). The relationship between exercise capacity and different functional markers in pulmonary rehabilitation for COPD. *Int. J. Chron. Obstruct. Pulmon. Dis.* 13 717–724. 10.2147/copd.s153525 29535512 PMC5836697

[B16] Kolaczkowska E. Kubes P. (2013). Neutrophil recruitment and function in health and inflammation. *Nat. Rev. Immunol.* 13 159–175. 10.1038/nri3399 23435331

[B17] Lacerda A. C. Marubayashi U. Coimbra C. C. (2005). Nitric oxide pathway is an important modulator of heat loss in rats during exercise. *Brain Res. Bull.* 67 110–116. 10.1016/j.brainresbull.2005.06.002 16140169

[B18] Medzhitov R. (2010). Inflammation 2010: new adventures of an old flame. *Cell* 140 771–776. 10.1016/j.cell.2010.03.006 20303867

[B19] Nieman D. C. Nehlsen-Cannarella S. L. Fagoaga O. R. Henson D. A. Utter A. Davis J. M. (1998). Influence of mode and carbohydrate on the cytokine response to heavy exertion. *Med. Sci. Sports Exerc.* 30 671–678. 10.1097/00005768-199805000-00005 9588607

[B20] Nunes-Silva A. Bernardes P. T. Rezende B. M. Lopes F. Gomes E. C. Marques P. E. (2014). Treadmill exercise induces neutrophil recruitment into muscle tissue in a reactive oxygen species-dependent manner: an intravital microscopy study. *PLoS One* 9:e96464. 10.1371/journal.pone.0096464 24798414 PMC4010495

[B21] Ogilvie R. W. Armstrong R. B. Baird K. E. Bottoms C. L. (1988). Lesions in the rat soleus muscle following eccentrically biased exercise. *Am. J. Anat.* 182 335–346. 10.1002/aja.1001820405 3189194

[B22] Ostrowski K. Rohde T. Asp S. Schjerling P. Pedersen B. K. (2001). Chemokines are elevated in plasma after strenuous exercise in humans. *Eur. J. Appl. Physiol.* 84 244–245. 10.1007/s004210170012 11320643

[B23] Pedersen B. K. Saltin B. (2015). Exercise as medicine - evidence for prescribing exercise as therapy in 26 different chronic diseases. *Scand. J. Med. Sci. Sports* 25(Suppl. 3), 1–72. 10.1111/sms.12581 26606383

[B24] Petersen A. M. Pedersen B. K. (2006). The role of IL-6 in mediating the anti-inflammatory effects of exercise. *J. Physiol. Pharmacol.* 57(Suppl. 10), 43–51. 10.1249/00005768-200605001-00226 17242490

[B25] Primola-Gomes T. N. Pires W. Rodrigues L. O. Coimbra C. C. Marubayashi U. Lima N. R. (2007). Activation of the central cholinergic pathway increases post-exercise tail heat loss in rats. *Neurosci. Lett.* 413 1–5. 10.1016/j.neulet.2006.10.042 17250962

[B26] Rezende B. M. Athayde R. M. Goncalves W. A. Resende C. B. Teles De Toledo Bernardes P. Perez D. A. (2017). Inhibition of 5-lipoxygenase alleviates graft-versus-host disease. *J. Exp. Med.* 214 3399–3415. 28947611 10.1084/jem.20170261 PMC5679175

[B27] Rizo-Roca D. Rios-Kristjansson J. G. Nunez-Espinosa C. Ascensao A. Magalhaes J. Torrella J. R. (2015). A semiquantitative scoring tool to evaluate eccentric exercise-induced muscle damage in trained rats. *Eur. J. Histochem.* 59:2544. 10.4081/ejh.2015.2544 PMC4698611 26708179

[B28] Rothschild J. A. Bishop D. J. (2020). Effects of dietary supplements on adaptations to endurance training. *Sports Med.* 50 25–53. 10.1007/s40279-019-01185-8 31531769

[B29] Russo R. C. Savino B. Mirolo M. Buracchi C. Germano G. Anselmo A. (2018). The atypical chemokine receptor ACKR2 drives pulmonary fibrosis by tuning influx of CCR2(+) and CCR5(+) IFNgamma-producing gammadeltaT cells in mice. *Am. J. Physiol. Lung Cell Mol. Physiol.* 314 L1010–L1025. 29469612 10.1152/ajplung.00233.2017

[B30] Saini J. Mcphee J. S. Al-Dabbagh S. Stewart C. E. Al-Shanti N. (2016). Regenerative function of immune system: modulation of muscle stem cells. *Age. Res. Rev.* 27 67–76. 10.1016/j.arr.2016.03.006 27039885

[B31] Serrano A. L. Baeza-Raja B. Perdiguero E. Jardi M. Munoz-Canoves P. (2008). Interleukin-6 is an essential regulator of satellite cell-mediated skeletal muscle hypertrophy. *Cell Metab.* 7 33–44. 10.1016/j.cmet.2007.11.011 18177723

[B32] Summers C. Rankin S. M. Condliffe A. M. Singh N. Peters A. M. Chilvers E. R. (2010). Neutrophil kinetics in health and disease. *Trends Immunol.* 31 318–324. 10.1016/j.it.2010.05.006 20620114 PMC2930213

[B33] Suzuki K. Naganuma S. Totsuka M. Suzuki K. J. Mochizuki M. Shiraishi M. (1996). Effects of exhaustive endurance exercise and its one-week daily repetition on neutrophil count and functional status in untrained men. *Int. J. Sports Med.* 17 205–212. 10.1055/s-2007-972833 8739575

[B34] Suzuki K. Nakaji S. Yamada M. Totsuka M. Sato K. Sugawara K. (2002). Systemic inflammatory response to exhaustive exercise, Cytokine kinetics. *Exerc. Immunol. Rev.* 8 6–48. 12690937

[B35] Suzuki K. Totsuka M. Nakaji S. Yamada M. Kudoh S. Liu Q. (1999). Endurance exercise causes interaction among stress hormones, cytokines, neutrophil dynamics, and muscle damage. *J. Appl. Physiol.* 87 1360–1367. 10.1152/jappl.1999.87.4.1360 10517764

[B36] Suzuki K. Yamada M. Kurakake S. Okamura N. Yamaya K. Liu Q. (2000). Circulating cytokines and hormones with immunosuppressive but neutrophil-priming potentials rise after endurance exercise in humans. *Eur. J. Appl. Physiol.* 81 281–287. 10.1007/s004210050044 10664086

[B37] Taherkhani S. Suzuki K. Castell L. (2020). A short overview of changes in inflammatory cytokines and oxidative stress in response to physical activity and antioxidant supplementation. *Antioxidants* 9:886. 10.3390/antiox9090886 32962110 PMC7555806

[B38] Tavares-Murta B. M. Zaparoli M. Ferreira R. B. Silva-Vergara M. L. Oliveira C. H. Murta E. F. (2002). Failure of neutrophil chemotactic function in septic patients. *Crit. Care Med.* 30 1056–1061. 10.1097/00003246-200205000-00017 12006803

[B39] Yamada M. Suzuki K. Kudo S. Totsuka M. Nakaji S. Sugawara K. (2002). Raised plasma G-CSF and IL-6 after exercise may play a role in neutrophil mobilization into the circulation. *J. Appl. Physiol.* 92 1789–1794. 10.1152/japplphysiol.00629.2001 11960925

