# Supplementary figures and images for: Effect of Physical Training on Exercise-Induced Inflammation and Performance in Mice
Source: Front Cell Dev Biol. 2021 Feb 4;9:625680. doi: 10.3389/fcell.2021.625680 (PMC7891665; doi:10.3389/fcell.2021.625680)

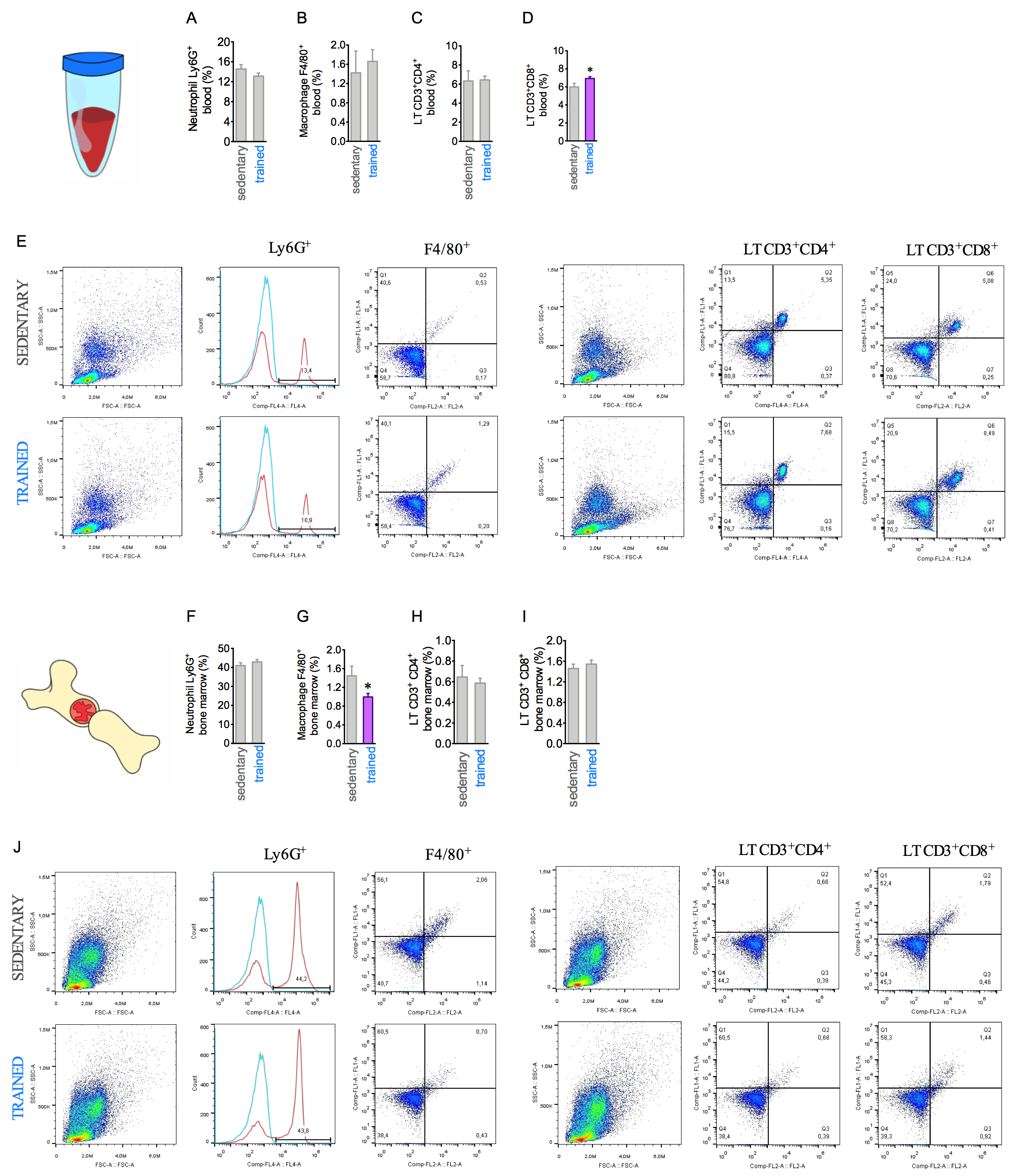

Supplement: Supplementary Figure 1 — Effect of aerobic training on the profile of immune cells. The percentage of neutrophil Ly6G+, macrophage F480+, TCD4+ and TCD8+ cells of the sedentary (n = 7) and training (n = 8) mice were evaluated in blood (A–D, respectively) and bone marrow (F–I, respectively) by flow cytometry 72 h after fixed-speed running test until fatigue post training. Representative dot plots illustrating neutrophil Ly6G+, macrophage F480+, TCD4+, and TCD8+ cells of the sedentary (upper panel) and trained (below panel) mice in the blood (E) and bone marrow (J). Data are expressed as mean ± SEM; ∗p < 0.05 compared to sedentary using two-tailed t-test. Cells from bone marrow and blood were plated, 1 × 10^6^ cells/well, in a 96-well plate and stained for extracellular molecular expression patterns using mAbs against mouse CD3 (Alexa-488 conjugated), CD4 (APC conjugated), CD8 (PE conjugated), CD11b (Alexa-488 conjugated), F4/80 (PE conjugated), and Ly6G (APC conjugated) (from BD Pharmingen, Le Pont de Claix, France). The cells were incubated with 20 μl/well of antibody solution 30’/4°C, followed by fixation in 4% of paraformaldehyde. Limits for the quadrant markers were always set based on negative populations and isotype control antibodies. The frequency of positive cells was analyzed by FlowJo X v10.2 software, using a gate that included lymphocytes, neutrophils and/or macrophages. The frequency (percentage) of the analyzed population in the total acquired events was used in the construction of the graphs. [file Image_1.TIFF]
